# Supplementary material for: High-Pressure Processing Influences Antibiotic Resistance Gene Transfer in Listeria monocytogenes Isolated from Food and Processing Environments
Source: Int J Mol Sci. 2024 Dec 2;25(23):12964. doi: 10.3390/ijms252312964 (PMC11641147; doi:10.3390/ijms252312964)
Supplement: Supplementary file 1 [file ijms-25-12964-s001.zip › ijms-3321299-supplementary.pdf]

Table S1. Results of gene transfer in microbial culture medium (*in vitro*) and food matrix (*in situ*) before and after exposure to HPP.

Antibiotic	TC	Microbial culture medium (*in vitro*)			Food matrix (*in situ*)		
		Control	200 MPa	Recovery after 400 MPa	Control	200 MPa	Recovery after 400 MPa
		TC/R±SD					
TET	TC-Lm_1	4.74±0.14 x 10^{-1}	4.73±0.14 x 10^{-1}	0.11±0.01 x 10^{1}	4.91±0.07 x 10^{-1}	4.74±0.14 x 10^{-1}	1.51±0.03 x 10^{2}
	TC-Lm_2	0.25±0.01 x 10^{1}	0.24±0.01 x 10^{1}	0.27±0.07 x 10^{1}	2.26±0.05 x 10^{-1}	0.25±0.01 x 10^{1}	0.12±0.01 x 10^{1}
	TC-Lm_3	3.53±0.03 x 10^{-1}	3.51±0.04 x 10^{-1}	0.16±0.00 x 10^{1}	2.30±0.09 x 10^{-1}	3.55±0.16 x 10^{-1}	0.70±0.02 x 10^{-1}
	TC-Lm_4	1.29±0.02 x 10^{-1}	1.30±0.01 x 10^{-1}	3.40±0.11 x 10^{-1}	5.69±0.05 x 10^{-1}	1.31±0.02 x 10^{-1}	0.41±0.02 x 10^{1}
	TC-Lm_5	5.21±0.06 x 10^{-2}	5.19±0.09 x 10^{-2}	0.24±0.01 x 10^{-1}	4.71±0.07 x 10^{-1}	5.39±0.16 x 10^{-2}	0.13±0.01 x 10^{1}
	TC-Lm_6	8.00±0.06 x 10^{-1}	7.97±0.06 x 10^{-1}	0.11±0.01 x 10^{-1}	4.71±0.08 x 10^{-1}	8.03±0.05 x 10^{-1}	0.52±0.01 x 10^{1}
LIN	TC-Lm_1	3.01±0.02 x 10^{-1}	3.06±0.04 x 10^{-1}	8.25±0.08 x 10^{-1}	4.79±0.04 x 10^{-1}	2.94±0.06 x 10^{-1}	0.18±0.01 x 10^{1}
	TC-Lm_2	4.01±0.01 x 10^{-1}	4.05±0.06 x 10^{-1}	3.51±0.07 x 10^{-1}	2.12±0.13 x 10^{-1}	3.95±0.05 x 10^{-1}	6.81±0.10 x 10^{-1}
	TC-Lm_3	1.97±0.02 x 10^{-1}	1.99±0.04 x 10^{-1}	0.10±0.00 x 10^{1}	2.17±0.07 x 10^{-1}	2.02±0.03 x 10^{-1}	3.38±0.07 x 10^{-1}
	TC-Lm_4	3.19±0.03 x 10^{-1}	3.18±0.03 x 10^{-1}	0.10±0.00 x 10^{1}	1.93±0.02 x 10^{-1}	3.18±0.04 x 10^{-1}	0.19±0.01 x 10^{1}
	TC-Lm_5	2.10±0.01 x 10^{-1}	2.14±0.04 x 10^{-1}	7.71±0.17 x 10^{-1}	5.19±0.10 x 10^{-1}	2.05±0.04 x 10^{-1}	0.10±0.00 x 10^{-1}
	TC-Lm_6	6.99±0.08 x 10^{-1}	7.00±0.04 x 10^{-1}	0.11±0.01 x 10^{1}	4.62±0.07 x 10^{-1}	6.98±0.07 x 10^{-1}	0.12±0.00 x 10^{-1}
CIP	TC-Lm_1	0.34±0.00 x 10^{1}	0.34±0.00 x 10^{1}	0.14±0.00 x 10^{1}	2.56±0.03 x 10^{-1}	0.34±0.01 x 10^{1}	8.51±0.06 x 10^{1}
	TC-Lm_2	0.26±0.00 x 10^{1}	0.26±0.01 x 10^{1}	6.76±0.30 x 10^{-1}	1.53±0.02 x 10^{-3}	0.27±0.01 x 10^{1}	6.46±0.02 x 10^{1}
	TC-Lm_3	2.70±0.01 x 10^{-1}	2.69±0.01 x 10^{-1}	8.73±0.39 x 10^{-1}	3.53±0.32 x 10^{-3}	2.65±0.08 x 10^{-1}	2.55±0.10 x 10^{1}
	TC-Lm_4	6.15±0.05 x 10^{-1}	6.11±0.10 x 10^{-1}	1.51±0.04 x 10^{-1}	1.51±0.18 x 10^{-1}	6.16±0.06 x 10^{-1}	1.48±0.02 x 10^{2}
	TC-Lm_5	7.30±0.02 x 10^{-1}	6.92±0.32 x 10^{-1}	0.14±0.01 x 10^{1}	7.66±0.03 x 10^{-2}	7.08±0.19 x 10^{-1}	5.99±0.01 x 10^{1}
	TC-Lm_6	1.36±0.04 x 10^{-1}	1.38±0.01 x 10^{-1}	4.59±0.06 x 10^{-1}	1.81±0.01 x 10^{-2}	1.37±0.03 x 10^{-1}	5.65±0.21 x 10^{2}
FOS	TC-Lm_1	3.29±0.03 x 10^{-1}	3.36±0.09 x 10^{-1}	0.11±0.00 x 10^{1}	3.27±0.05 x 10^{-1}	3.34±0.09 x 10^{-1}	7.47±0.13 x 10^{-1}
	TC-Lm_2	3.48±0.06 x 10^{-1}	3.51±0.05 x 10^{-1}	0.12±0.00 x 10^{1}	2.45±0.04 x 10^{-1}	3.51±0.05 x 10^{-1}	8.84±0.20 x 10^{-1}
	TC-Lm_3	2.96±0.01 x 10^{-1}	3.03±0.07 x 10^{-1}	0.15±0.00 x 10^{1}	0.45±0.00 x 10^{1}	2.83±0.28 x 10^{-1}	4.87±0.04 x 10^{-1}
	TC-Lm_4	2.25±0.04 x 10^{-1}	2.31±0.10 x 10^{-1}	0.12±0.01 x 10^{1}	5.64±0.05 x 10^{-1}	2.29±0.08 x 10^{-1}	0.98±0.02 x 10^{-1}
	TC-Lm_5	4.95±0.04 x 10^{-1}	4.92±0.02 x 10^{-1}	9.49±0.20 x 10^{-1}	1.46±0.04 x 10^{-1}	5.09±0.19 x 10^{-1}	7.17±0.18 x 10^{-1}
	TC-Lm_6	0.27±0.00 x 10^{1}	2.68±0.02 x 10^{1}	0.17±0.01 x 10^{1}	1.74±0.02 x 10^{-1}	0.27±0.00 x 10^{-1}	7.36±0.02 x 10^{-1}
DA	TC-Lm_1	3.47±0.03 x 10^{-1}	3.43±0.05 x 10^{-1}	9.54±0.36 x 10^{-1}	6.00±0.00 x 10^{-1}	3.49±0.03 x 10^{-1}	0.10±0.00 x 10^{-1}
	TC-Lm_2	5.24±0.08 x 10^{-1}	5.19±0.03 x 10^{-1}	3.15±0.10 x 10^{-1}	6.77±0.03 x 10^{-1}	5.23±0.05 x 10^{-1}	6.43±0.05 x 10^{-1}
	TC-Lm_3	4.11±0.07 x 10^{-1}	4.10±0.05 x 10^{-1}	0.40±0.51 x 10^{1}	8.47±0.46 x 10^{-2}	4.05±0.02 x 10^{-1}	5.54±0.07 x 10^{-1}
	TC-Lm_4	3.13±0.02 x 10^{-1}	3.17±0.04 x 10^{-1}	8.94±0.09 x 10^{-1}	5.12±0.04 x 10^{-1}	3.17±0.03 x 10^{-1}	0.16±0.00 x 10^{1}
	TC-Lm_5	0.19±0.00 x 10^{1}	1.92±0.02 x 10^{-1}	8.53±0.19 x 10^{-1}	0.10±0.00 x 10^{1}	0.19±0.00 x 10^{-1}	0.12±0.00 x 10^{-1}
	TC-Lm_6	0.37±0.01 x 10^{1}	0.38±0.01 x 10^{1}	8.56±0.30 x 10^{-1}	6.87±0.05 x 10^{-1}	0.37±0.01 x 10^{1}	8.22±0.05 x 10^{-1}

Abbreviations: Green – a decrease in the frequency of gene transfer compared to the control value; Red – increase in gene transfer frequency compared to control value; TC – transconjugant; TC/R – transfer rate, TET – tetracycline, LIN – lincomycin, CIP – ciprofloxacin, FOS – fosfomycin, DA – clindamycin; Control – control sample (transconjugant obtained from the strain before HPP treatment); 200 MPa – transconjugant obtained from the strain after exposure to 200 MPa pressure; 400 MPa – transconjugant obtained from the recovered strain after exposure to 400 MPa pressure.

Table S2. Antibiotic resistance gene transfer in *Listeria monocytogenes* strains before exposure to HPP in microbial culture medium (*in vitro*).

		**T-BHI**	**TD-BHI**	**B-BHI**	**TC/R**	**SD – TC/R**	**Wyniki**
		TC – transconjugants	R – recipient	D - donor	Transfer Rate	±	TC/R±SD
**TET**	**Lm_1**	9.78 x 10^{8}	2.00 x 10^{9}	2.12 x 10^{9}	4.89 x 10^{-1}	0.14	4.74±0.14 x 10^{-1}
		9.70 x 10^{8}	2.10 x 10^{9}	2.10 x 10^{9}	4.62 x 10^{-1}		
		9.79 x 10^{8}	2.08 x 10^{9}	2.11 x 10^{9}	4.71 x 10^{-1}		
	**Lm_2**	5.52 x 10^{8}	2.20 x 10^{8}	4.40 x 10^{8}	0.25 x 10^{1}	0.01	0.25±0.01 x 10^{1}
		5.50 x 10^{8}	2.22 x 10^{8}	4.41 x 10^{8}	0.25 x 10^{1}		
		5.51 x 10^{8}	2.22 x 10^{8}	4.43 x 10^{8}	0.24 x 10^{1}		
	**Lm_3**	3.80 x 10^{8}	1.07 x 10^{9}	1.32 x 10^{9}	3.55 x 10^{-1}	0.03	3.53±0.03 x 10^{-1}
		3.82 x 10^{8}	1.09 x 10^{8}	1.33 x 10^{9}	3.50 x 10^{-1}		
		3.85 x 10^{8}	1.09 x 10^{8}	1.34 x 10^{9}	3.53 x 10^{-1}		
	**Lm_4**	3.36 x 10^{8}	2.60 x 10^{9}	3.20 x 10^{9}	1.29 x 10^{-1}	0.02	1.29±0.02 x 10^{-1}
		3.30 x 10^{8}	2.59 x 10^{9}	3.21 x 10^{9}	1.27 x 10^{-1}		
		3.38 x 10^{8}	2.60 x 10^{9}	3.24 x 10^{9}	1.30 x 10^{-1}		
	**Lm_5**	6.56 x 10^{8}	1.26 x 10^{10}	7.56 x 10^{9}	5.21 x 10^{-2}	0.06	5.21±0.06 x 10^{-2}
		6.55 x 10^{8}	1.27 x 10^{10}	7.57 x 10^{9}	5.15 x 10^{-2}		
		6.58 x 10^{8}	1.25 x 10^{10}	7.59 x 10^{9}	5.26 x 10^{-2}		
	**Lm_6**	4.80 x 10^{8}	6.00 x 10^{8}	7.40 x 10^{8}	8.00 x 10^{-1}	0.06	8.00±0.06 x 10^{-1}
		4.85 x 10^{8}	6.10 x 10^{8}	7.41 x 10^{8}	7.95 x 10^{-1}		
		4.85 x 10^{8}	6.02 x 10^{8}	7.45 x 10^{8}	8.06 x 10^{-1}		
**LIN**	**Lm_1**	5.52 x 10^{8}	1.83 x 10^{9}	1.12 x 10^{9}	4.89 x 10^{-1}	0.02	3.01±0.02 x 10^{-1}
		5.54 x 10^{8}	1.85 x 10^{9}	2.40 x 10^{9}	4.62 x 10^{-1}		
		5.55 x 10^{8}	1.84 x 10^{9}	2.51 x 10^{9}	4.71 x 10^{-1}		
	**Lm_2**	1.48 x 10^{8}	3.70 x 10^{8}	4.90 x 10^{8}	0.25 x 10^{1}	0.01	4.01±0.01 x 10^{-1}
		1.49 x 10^{8}	3.71 x 10^{8}	4.81 x 10^{8}	0.25 x 10^{1}		
		1.50 x 10^{8}	3.75 x 10^{8}	4.73 x 10^{8}	0.24 x 10^{1}		
	**Lm_3**	3.88 x 10^{8}	1.95 x 10^{9}	1.42 x 10^{9}	3.55 x 10^{-1}	0.02	1.97±0.02 x 10^{-1}
		3.89 x 10^{8}	1.99 x 10^{9}	1.33 x 10^{9}	3.50 x 10^{-1}		
		3.85 x 10^{8}	1.95 x 10^{9}	1.24 x 10^{9}	3.53 x 10^{-1}		
	**Lm_4**	6.68 x 10^{8}	2.08 x 10^{9}	3.70 x 10^{9}	1.29 x 10^{-1}	0.03	3.19±0.03 x 10^{-1}
		6.69 x 10^{8}	2.09 x 10^{9}	3.41 x 10^{9}	1.27 x 10^{-1}		
		6.66 x 10^{8}	2.11 x 10^{9}	3.54 x 10^{9}	1.30 x 10^{-1}		
	**Lm_5**	1.24 x 10^{9}	5.93 x 10^{9}	5.56 x 10^{9}	5.21 x 10^{-2}	0.01	2.10±0.01 x 10^{-1}
		1.25 x 10^{9}	5.94 x 10^{9}	5.77 x 10^{9}	5.15 x 10^{-2}		
		1.25 x 10^{9}	5.96 x 10^{9}	5.79 x 10^{9}	5.26 x 10^{-2}		
	**Lm_6**	2.52 x 10^{8}	3.60 x 10^{8}	6.50 x 10^{8}	8.00 x 10^{-1}	0.08	6.99±0.08 x 10^{-1}
		2.55 x 10^{8}	3.61 x 10^{8}	6.51 x 10^{8}	7.95 x 10^{-1}		
		2.51 x 10^{8}	3.63 x 10^{8}	6.65 x 10^{8}	8.06 x 10^{-1}		
**CIP**	**Lm_1**	5.46 x 10^{8}	1.60 x 10^{8}	1.12 x 10^{8}	4.89 x 10^{-1}	0.00	0.34±0.00 x 10^{1}
		5.43 x 10^{8}	1.62 x 10^{8}	1.10 x 10^{8}	4.62 x 10^{-1}		
		5.50 x 10^{8}	1.62 x 10^{8}	1.11 x 10^{8}	4.71 x 10^{-1}		
	**Lm_2**	2.60 x 10^{8}	9.92 x 10^{7}	4.90 x 10^{8}	0.25 x 10^{1}	0.00	0.26±0.00 x 10^{1}
		2.55 x 10^{8}	9.90 x 10^{7}	4.91 x 10^{8}	0.25 x 10^{1}		
		2.60 x 10^{8}	9.89 x 10^{7}	4.83 x 10^{8}	0.24 x 10^{1}		
	**Lm_3**	2.08 x 10^{8}	7.80 x 10^{8}	1.92 x 10^{9}	3.55 x 10^{-1}	0.01	2.70±0.01 x 10^{-1}
		2.09 x 10^{8}	7.77 x 10^{8}	1.93 x 10^{9}	3.50 x 10^{-1}		

		2.11×10^{8}	7.79×10^{8}	1.94×10^{9}	3.53×10^{-1}		
	**Lm_4**	2.80×10^{8}	4.60×10^{8}	2.60×10^{9}	1.29×10^{-1}	0.05	$6.15 \pm 0.05 \times 10^{-1}$
		2.85×10^{8}	4.62×10^{8}	2.61×10^{9}	1.27×10^{-1}		
		2.86×10^{8}	4.62×10^{8}	2.64×10^{9}	1.30×10^{-1}		
	**Lm_5**	7.36×10^{8}	1.01×10^{9}	8.32×10^{9}	5.21×10^{-2}	0.02	$7.30 \pm 0.02 \times 10^{-1}$
		7.33×10^{8}	1.00×10^{9}	8.27×10^{9}	5.15×10^{-2}		
		7.36×10^{8}	1.01×10^{9}	8.29×10^{9}	5.26×10^{-2}		
	**Lm_6**	1.56×10^{8}	1.12×10^{9}	6.85×10^{8}	8.00×10^{-1}	0.04	$1.36 \pm 0.04 \times 10^{-1}$
		1.57×10^{8}	1.15×10^{9}	6.90×10^{8}	7.95×10^{-1}		
		1.57×10^{8}	1.19×10^{9}	6.92×10^{8}	8.06×10^{-1}		
**FOS**	**Lm_1**	1.13×10^{9}	3.46×10^{9}	2.72×10^{9}	3.27×10^{-1}	0.02	$3.29 \pm 0.03 \times 10^{-1}$
		1.15×10^{9}	3.48×10^{9}	2.77×10^{9}	3.30×10^{-1}		
		1.15×10^{9}	3.49×10^{9}	2.77×10^{9}	3.30×10^{-1}		
	**Lm_2**	9.28×10^{8}	2.62×10^{9}	2.39×10^{9}	3.54×10^{-1}	0.06	$3.48 \pm 0.06 \times 10^{-1}$
		9.29×10^{8}	2.69×10^{9}	2.30×10^{9}	3.45×10^{-1}		
		9.28×10^{8}	2.70×10^{9}	2.32×10^{9}	3.44×10^{-1}		
	**Lm_3**	1.35×10^{9}	4.57×10^{9}	2.34×10^{9}	2.95×10^{-1}	0.01	$2.96 \pm 0.01 \times 10^{-1}$
		1.36×10^{9}	4.59×10^{9}	2.36×10^{9}	2.96×10^{-1}		
		1.36×10^{9}	4.60×10^{9}	2.39×10^{9}	2.96×10^{-1}		
	**Lm_4**	1.03×10^{9}	4.69×10^{9}	3.38×10^{9}	2.20×10^{-1}	0.04	$2.25 \pm 0.04 \times 10^{-1}$
		1.05×10^{9}	4.60×10^{9}	3.41×10^{9}	2.28×10^{-1}		
		1.05×10^{9}	4.62×10^{9}	3.36×10^{9}	2.27×10^{-1}		
	**Lm_5**	1.07×10^{9}	2.18×10^{9}	2.10×10^{9}	4.91×10^{-1}	0.04	$4.95 \pm 0.04 \times 10^{-1}$
		1.09×10^{9}	2.19×10^{9}	2.12×10^{9}	4.98×10^{-1}		
		1.09×10^{9}	2.20×10^{9}	2.10×10^{9}	4.95×10^{-1}		
	**Lm_6**	8.55×10^{8}	3.13×10^{8}	1.83×10^{9}	0.27×10^{1}	0.00	$0.27 \pm 0.00 \times 10^{1}$
		8.55×10^{8}	3.15×10^{8}	1.83×10^{9}	0.27×10^{1}		
		8.56×10^{8}	3.13×10^{8}	1.87×10^{8}	0.27×10^{1}		
**DA**	**Lm_1**	7.84×10^{8}	2.24×10^{9}	1.32×10^{8}	3.27×10^{-1}	0.03	$3.47 \pm 0.03 \times 10^{-1}$
		7.85×10^{8}	2.26×10^{9}	1.37×10^{8}	3.30×10^{-1}		
		7.89×10^{8}	2.29×10^{9}	1.37×10^{9}	3.30×10^{-1}		
	**Lm_2**	8.72×10^{8}	1.67×10^{9}	2.39×10^{9}	3.54×10^{-1}	0.08	$5.24 \pm 0.08 \times 10^{-1}$
		8.74×10^{8}	1.69×10^{9}	2.30×10^{9}	3.45×10^{-1}		
		8.79×10^{8}	1.65×10^{9}	2.32×10^{9}	3.44×10^{-1}		
	**Lm_3**	7.20×10^{8}	1.78×10^{9}	4.64×10^{8}	2.95×10^{-1}	0.07	$4.11 \pm 0.07 \times 10^{-1}$
		7.22×10^{8}	1.75×10^{9}	4.56×10^{8}	2.96×10^{-1}		
		7.23×10^{8}	1.73×10^{9}	4.59×10^{8}	2.96×10^{-1}		
	**Lm_4**	9.28×10^{8}	2.95×10^{9}	3.38×10^{9}	2.20×10^{-1}	0.02	$3.13 \pm 0.02 \times 10^{-1}$
		9.30×10^{8}	2.99×10^{9}	3.41×10^{9}	2.28×10^{-1}		
		9.30×10^{8}	2.98×10^{9}	3.36×10^{9}	2.27×10^{-1}		
	**Lm_5**	5.64×10^{8}	2.98×10^{8}	6.40×10^{9}	4.91×10^{-1}	0.00	$0.19 \pm 0.00 \times 10^{1}$
		5.65×10^{8}	2.95×10^{8}	6.42×10^{9}	4.98×10^{-1}		
		5.65×10^{8}	2.96×10^{8}	6.50×10^{9}	4.95×10^{-1}		
	**Lm_6**	6.00×10^{8}	1.60×10^{8}	5.23×10^{8}	0.27×10^{1}	0.01	$0.37 \pm 0.01 \times 10^{1}$
		6.01×10^{8}	1.63×10^{8}	5.33×10^{8}	0.27×10^{1}		
		6.03×10^{8}	1.64×10^{8}	5.27×10^{8}	0.27×10^{1}		

TET – tetracycline, LIN – lincomycin, CIP – ciprofloxacin, FOS – fosfomycin, DA – clindamycin.

Table S3. Antibiotic resistance gene transfer in *Listeria monocytogenes* strains before exposure to HPP in food matrix (*in situ*).

		T-BHI	TD-BHI	B-BHI	TC/R	SD – TC/R	Wyniki
		TC – transconjugants	R – recipient	D - donor	Transfer Rate	±	TC/R±SD
TET	Lm_1	3.28×10^8	6.60×10^8	1.11×10^9	4.97×10^{-1}	0.07	$4.91 \pm 0.07 \times 10^{-1}$
		3.20×10^8	6.65×10^8	1.19×10^9	4.81×10^{-1}		
		3.30×10^8	6.68×10^8	1.20×10^9	4.94×10^{-1}		
	Lm_2	3.68×10^8	1.64×10^9	1.86×10^9	2.24×10^{-1}	0.05	$2.26 \pm 0.05 \times 10^{-1}$
		3.72×10^8	1.60×10^9	1.89×10^9	2.33×10^{-1}		
		3.70×10^8	1.67×10^9	1.79×10^9	2.22×10^{-1}		
	Lm_3	2.80×10^8	1.21×10^9	1.30×10^9	2.32×10^{-1}	0.09	$2.30 \pm 0.09 \times 10^{-1}$
		2.85×10^8	1.19×10^9	1.39×10^9	2.39×10^{-1}		
		2.77×10^8	1.27×10^9	1.39×10^9	2.18×10^{-1}		
	Lm_4	4.40×10^8	7.70×10^8	1.18×10^9	5.71×10^{-1}	0.05	$5.69 \pm 0.05 \times 10^{-1}$
		4.45×10^8	7.74×10^8	1.18×10^9	5.75×10^{-1}		
		4.38×10^8	7.79×10^8	1.20×10^9	5.62×10^{-1}		
	Lm_5	2.13×10^8	4.60×10^8	8.00×10^8	4.63×10^{-1}	0.07	$4.71 \pm 0.07 \times 10^{-1}$
		2.18×10^8	4.65×10^8	8.21×10^9	4.69×10^{-1}		
		2.19×10^8	4.55×10^8	8.10×10^9	4.81×10^{-1}		
	Lm_6	3.18×10^8	6.90×10^8	1.52×10^9	4.61×10^{-1}	0.08	$4.71 \pm 0.08 \times 10^{-1}$
		3.25×10^8	6.88×10^8	1.59×10^9	4.72×10^{-1}		
		3.27×10^8	6.80×10^8	1.59×10^9	4.81×10^{-1}		
LIN	Lm_1	4.56×10^8	9.60×10^8	2.12×10^8	4.97×10^{-1}	0.04	$4.79 \pm 0.04 \times 10^{-1}$
		4.60×10^8	9.65×10^8	2.17×10^8	4.81×10^{-1}		
		4.65×10^8	9.58×10^8	2.17×10^9	4.94×10^{-1}		
	Lm_2	3.86×10^8	1.72×10^9	2.89×10^9	2.24×10^{-1}	0.13	$2.12 \pm 0.13 \times 10^{-1}$
		3.87×10^8	1.78×10^9	2.80×10^9	2.33×10^{-1}		
		3.42×10^8	1.76×10^9	2.82×10^9	2.22×10^{-1}		
	Lm_3	2.44×10^8	1.12×10^9	4.04×10^8	2.32×10^{-1}	0.07	$2.17 \pm 0.07 \times 10^{-1}$
		2.44×10^8	1.08×10^9	4.06×10^8	2.39×10^{-1}		
		2.48×10^8	1.19×10^9	4.03×10^8	2.18×10^{-1}		
	Lm_4	2.76×10^8	1.41×10^9	2.88×10^9	5.71×10^{-1}	0.02	$1.93 \pm 0.02 \times 10^{-1}$
		2.77×10^8	1.46×10^9	2.81×10^9	5.75×10^{-1}		
		2.80×10^8	1.46×10^9	2.86×10^9	5.62×10^{-1}		
	Lm_5	2.68×10^8	5.10×10^8	2.45×10^9	4.63×10^{-1}	0.10	$5.19 \pm 0.10 \times 10^{-1}$
		2.69×10^8	5.10×10^8	2.48×10^9	4.69×10^{-1}		
		2.60×10^8	5.15×10^8	2.50×10^9	4.81×10^{-1}		
	Lm_6	5.44×10^8	1.10×10^9	8.23×10^8	4.61×10^{-1}	0.07	$4.62 \pm 0.07 \times 10^{-1}$
		5.45×10^8	1.19×10^9	8.33×10^8	4.72×10^{-1}		
		5.43×10^8	1.15×10^9	8.27×10^8	4.81×10^{-1}		
CIP	Lm_1	1.68×10^6	6.60×10^8	1.52×10^9	4.97×10^{-1}	0.03	$2.56 \pm 0.03 \times 10^{-1}$
		1.70×10^6	6.68×10^8	1.55×10^9	4.81×10^{-1}		
		1.72×10^6	6.63×10^8	1.56×10^9	4.94×10^{-1}		
	Lm_2	2.52×10^6	1.64×10^9	1.49×10^9	2.24×10^{-1}	0.02	$153 \pm 0.02 \times 10^{-3}$
		2.53×10^6	1.69×10^9	1.40×10^9	2.33×10^{-1}		
		2.56×10^6	1.65×10^9	1.42×10^9	2.22×10^{-1}		
	Lm_3	4.72×10^6	1.21×10^9	3.50×10^9	2.32×10^{-1}	0.32	$3.53 \pm 0.32 \times 10^{-3}$
		4.75×10^6	1.25×10^9	3.56×10^9	2.39×10^{-1}		

		4.76×10^{6}	1.28×10^{9}	3.59×10^{9}	2.18×10^{-1}		
	**Lm_4**	1.04×10^{8}	7.70×10^{8}	3.68×10^{9}	5.71×10^{-1}	0.18	$1.51 \pm 0.18 \times 10^{-1}$
		1.10×10^{8}	7.75×10^{8}	3.60×10^{9}	5.75×10^{-1}		
		1.04×10^{8}	7.79×10^{8}	3.66×10^{9}	5.62×10^{-1}		
	**Lm_5**	4.60×10^{6}	6.00×10^{7}	2.80×10^{9}	4.63×10^{-1}	0.03	$7.66 \pm 0.03 \times 10^{-2}$
		4.65×10^{6}	6.10×10^{7}	2.82×10^{9}	4.69×10^{-1}		
		4.65×10^{6}	6.05×10^{7}	2.80×10^{9}	4.81×10^{-1}		
	**Lm_6**	1.11×10^{7}	6.20×10^{6}	1.55×10^{9}	4.61×10^{-1}	0.01	$1.81 \pm 0.01 \times 10^{-2}$
		1.14×10^{7}	6.25×10^{6}	1.55×10^{9}	4.72×10^{-1}		
		1.15×10^{7}	6.35×10^{6}	1.60×10^{9}	4.81×10^{-1}		
**FOS**	**Lm_1**	4.56×10^{8}	1.38×10^{9}	1.15×10^{9}	4.97×10^{-1}	0.05	$3.27 \pm 0.05 \times 10^{-1}$
		4.59×10^{8}	1.39×10^{9}	1.15×10^{9}	4.81×10^{-1}		
		4.58×10^{8}	1.43×10^{9}	1.19×10^{9}	4.94×10^{-1}		
	**Lm_2**	5.28×10^{8}	2.11×10^{9}	6.10×10^{8}	2.24×10^{-1}	0.04	$2.45 \pm 0.04 \times 10^{-1}$
		5.30×10^{8}	2.17×10^{9}	6.11×10^{8}	2.33×10^{-1}		
		5.31×10^{8}	2.20×10^{9}	6.19×10^{8}	2.22×10^{-1}		
	**Lm_3**	3.56×10^{8}	7.92×10^{7}	2.62×10^{9}	2.32×10^{-1}	0.00	$0.45 \pm 0.00 \times 10^{1}$
		3.59×10^{8}	7.99×10^{7}	2.69×10^{9}	2.39×10^{-1}		
		3.59×10^{8}	7.90×10^{7}	2.64×10^{9}	2.18×10^{-1}		
	**Lm_4**	5.52×10^{8}	9.90×10^{8}	3.90×10^{9}	5.71×10^{-1}	0.05	$5.64 \pm 0.05 \times 10^{-1}$
		5.59×10^{8}	9.89×10^{8}	3.81×10^{9}	5.75×10^{-1}		
		5.60×10^{8}	9.85×10^{8}	3.84×10^{9}	5.62×10^{-1}		
	**Lm_5**	2.24×10^{8}	1.59×10^{9}	2.86×10^{9}	4.63×10^{-1}	0.04	$1.46 \pm 0.04 \times 10^{-1}$
		2.29×10^{8}	1.55×10^{9}	2.88×10^{9}	4.69×10^{-1}		
		2.29×10^{8}	1.53×10^{9}	2.94×10^{9}	4.81×10^{-1}		
	**Lm_6**	3.56×10^{8}	2.01×10^{9}	8.78×10^{8}	4.61×10^{-1}	0.02	$1.74 \pm 0.02 \times 10^{-1}$
		3.59×10^{8}	2.08×10^{9}	8.70×10^{8}	4.72×10^{-1}		
		3.55×10^{8}	2.06×10^{9}	8.70×10^{8}	4.81×10^{-1}		
**DA**	**Lm_1**	4.08×10^{8}	6.80×10^{8}	1.12×10^{9}	4.97×10^{-1}	0.00	$6.00 \pm 0.00 \times 10^{-1}$
		4.10×10^{8}	6.83×10^{8}	2.40×10^{9}	4.81×10^{-1}		
		4.10×10^{8}	6.85×10^{8}	2.51×10^{9}	4.94×10^{-1}		
	**Lm_2**	5.00×10^{8}	7.40×10^{8}	4.90×10^{8}	2.24×10^{-1}	0.03	$6.77 \pm 0.03 \times 10^{-1}$
		5.01×10^{8}	7.44×10^{8}	4.81×10^{8}	2.33×10^{-1}		
		5.08×10^{8}	7.46×10^{8}	4.73×10^{8}	2.22×10^{-1}		
	**Lm_3**	1.11×10^{8}	1.39×10^{9}	1.42×10^{9}	2.32×10^{-1}	0.46	$8.47 \pm 0.46 \times 10^{-2}$
		1.15×10^{8}	1.38×10^{9}	1.33×10^{9}	2.39×10^{-1}		
		1.18×10^{8}	1.30×10^{9}	1.24×10^{9}	2.18×10^{-1}		
	**Lm_4**	3.78×10^{8}	7.40×10^{8}	3.70×10^{9}	5.71×10^{-1}	0.04	$5.12 \pm 0.04 \times 10^{-1}$
		3.80×10^{8}	7.35×10^{8}	3.41×10^{9}	5.75×10^{-1}		
		3.76×10^{8}	7.40×10^{8}	3.54×10^{9}	5.62×10^{-1}		
	**Lm_5**	5.12×10^{8}	5.10×10^{8}	5.56×10^{9}	4.63×10^{-1}	0.00	$0.10 \pm 0.00 \times 10^{1}$
		5.12×10^{8}	5.13×10^{8}	5.77×10^{9}	4.69×10^{-1}		
		5.20×10^{8}	5.13×10^{8}	5.79×10^{9}	4.81×10^{-1}		
	**Lm_6**	5.24×10^{8}	7.60×10^{8}	6.50×10^{8}	4.61×10^{-1}	0.05	$6.87 \pm 0.05 \times 10^{-1}$
		5.29×10^{8}	7.65×10^{8}	6.51×10^{8}	4.72×10^{-1}		
		5.20×10^{8}	7.65×10^{8}	6.65×10^{8}	4.81×10^{-1}		

TET – tetracycline, LIN – lincomycin, CIP – ciprofloxacin, FOS – fosfomycin, DA – clindamycin.

Table S4. Antibiotic resistance gene transfer in *Listeria monocytogenes* strains after exposure to HPP (**200 MPa)** in microbial culture medium (*in vitro*).

		**T-BHI**	**TD-BHI**	**B-BHI**	**TC/R**	**SD – TC/R**	**Wyniki**
		TC – transconjugants	R – recipient	D - donor	Transfer Rate	±	TC/R±SD
**TET**	**Lm_1**	9.78 x 10^{8}	2.00 x 10^{9}	2.12 x 10^{9}	4.89 x 10^{-1}	0.14	4.73±0.14 x 10^{-1}
		9.70 x 10^{8}	2.09 x 10^{9}	2.15 x 10^{9}	4.64 x 10^{-1}		
		9.71 x 10^{8}	2.09 x 10^{9}	2.17 x 10^{9}	4.65 x 10^{-1}		
	**Lm_2**	5.52 x 10^{8}	2.20 x 10^{8}	4.40 x 10^{8}	0.25 x 10^{1}	0.01	0.24±0.01 x 10^{1}
		5.45 x 10^{8}	2.28 x 10^{8}	4.42 x 10^{8}	0.24 x 10^{1}		
		5.44 x 10^{8}	2.28 x 10^{8}	4.45 x 10^{8}	0.24 x 10^{1}		
	**Lm_3**	3.80 x 10^{8}	1.07 x 10^{9}	1.32 x 10^{9}	3.55 x 10^{-1}	0.04	3.51±0.04 x 10^{-1}
		3.89 x 10^{8}	1.12 x 10^{9}	1.38 x 10^{9}	3.47 x 10^{-1}		
		3.85 x 10^{8}	1.10 x 10^{9}	1.38 x 10^{9}	3.50 x 10^{-1}		
	**Lm_4**	3.36 x 10^{8}	2.60 x 10^{9}	3.20 x 10^{9}	1.29 x 10^{-1}	0.01	1.30±0.01 x 10^{-1}
		3.40 x 10^{8}	2.61 x 10^{9}	3.15 x 10^{9}	1.30 x 10^{-1}		
		3.41 x 10^{8}	2.63 x 10^{9}	3.15 x 10^{9}	1.30 x 10^{-1}		
	**Lm_5**	6.56 x 10^{8}	1.26 x 10^{10}	7.56 x 10^{9}	5.21 x 10^{-2}	0.09	5.19±0.09 x 10^{-2}
		6.58 x 10^{8}	1.29 x 10^{10}	7.54 x 10^{9}	5.10 x 10^{-2}		
		6.59 x 10^{8}	1.25 x 10^{10}	7.50 x 10^{9}	5.27 x 10^{-2}		
	**Lm_6**	4.80 x 10^{8}	6.00 x 10^{8}	7.40 x 10^{8}	8.00 x 10^{-1}	0.06	7.97±0.06 x 10^{-1}
		4.82 x 10^{8}	6.09 x 10^{8}	7.42 x 10^{8}	7.90 x 10^{-1}		
		4.85 x 10^{8}	6.05 x 10^{8}	7.46 x 10^{8}	8.02 x 10^{-1}		
**LIN**	**Lm_1**	5.52 x 10^{8}	1.83 x 10^{9}	2.12 x 10^{9}	3.01 x 10^{-1}	0.04	3.06±0.04 x 10^{-1}
		5.56 x 10^{8}	1.80 x 10^{9}	2.15 x 10^{9}	3.09 x 10^{-1}		
		5.60 x 10^{8}	1.82 x 10^{9}	2.17 x 10^{9}	3.08 x 10^{-1}		
	**Lm_2**	1.48 x 10^{8}	3.70 x 10^{8}	4.40 x 10^{8}	4.00 x 10^{-1}	0.06	4.05±0.06 x 10^{-1}
		1.50 x 10^{8}	3.73 x 10^{8}	4.42 x 10^{8}	4.02 x 10^{-1}		
		1.52 x 10^{8}	3.69 x 10^{8}	4.45 x 10^{8}	4.12 x 10^{-1}		
	**Lm_3**	3.88 x 10^{8}	1.95 x 10^{9}	1.32 x 10^{9}	1.99 x 10^{-1}	0.04	1.99±0.04 x 10^{-1}
		3.90 x 10^{8}	1.99 x 10^{9}	1.38 x 10^{9}	1.96 x 10^{-1}		
		3.92 x 10^{8}	1.93 x 10^{9}	1.38 x 10^{9}	2.03 x 10^{-1}		
	**Lm_4**	6.68 x 10^{8}	2.08 x 10^{9}	3.20 x 10^{9}	3.21 x 10^{-1}	0.03	3.18±0.03 x 10^{-1}
		6.70 x 10^{8}	2.12 x 10^{9}	3.15 x 10^{9}	3.16 x 10^{-1}		
		6.72 x 10^{8}	2.12 x 10^{9}	3.15 x 10^{9}	3.17 x 10^{-1}		
	**Lm_5**	1.24 x 10^{9}	5.93 x 10^{9}	7.56 x 10^{9}	2.10 x 10^{-1}	0.04	2.14±0.04 x 10^{-1}
		1.29 x 10^{9}	5.93 x 10^{9}	7.54 x 10^{9}	2.18 x 10^{-1}		
		1.29 x 10^{9}	5.99 x 10^{9}	7.50 x 10^{9}	2.15 x 10^{-1}		
	**Lm_6**	2.52 x 10^{8}	3.60 x 10^{8}	7.40 x 10^{8}	7.00 x 10^{-1}	0.04	7.00±0.04 x 10^{-1}
		2.55 x 10^{8}	3.62 x 10^{8}	7.42 x 10^{8}	7.04 x 10^{-1}		
		2.55 x 10^{8}	3.66 x 10^{8}	7.46 x 10^{8}	6.97 x 10^{-1}		
**CIP**	**Lm_1**	5.46 x 10^{8}	1.60 x 10^{8}	2.12 x 10^{9}	0.34 x 10^{1}	0.00	0.34±0.00 x 10^{1}
		5.47 x 10^{8}	1.63 x 10^{8}	2.15 x 10^{9}	0.34 x 10^{1}		
		5.49 x 10^{8}	1.64 x 10^{8}	2.17 x 10^{9}	0.34 x 10^{1}		
	**Lm_2**	2.60 x 10^{8}	9.92 x 10^{7}	4.40 x 10^{8}	0.26 x 10^{1}	0.01	0.26±0.01 x 10^{1}
		2.62 x 10^{8}	9.94 x 10^{7}	4.42 x 10^{8}	0.26 x 10^{1}		
		2.65 x 10^{8}	9.92 x 10^{7}	4.45 x 10^{8}	0.27 x 10^{1}		
	**Lm_3**	2.08 x 10^{8}	7.80 x 10^{8}	1.32 x 10^{9}	2.70 x 10^{-1}	0.01	2.69±0.01 x 10^{-1}
		2.10 x 10^{8}	7.82 x 10^{8}	1.38 x 10^{9}	2.69 x 10^{-1}		
		2.12 x 10^{8}	7.88 x 10^{8}	1.38 x 10^{9}	2.69 x 10^{-1}		

		2.80 x 10^{8}	4.60 x 10^{8}	3.20 x 10^{9}	6.10 x 10^{-1}		
	**Lm_4**	2.89 x 10^{8}	4.65 x 10^{8}	3.15 x 10^{9}	6.22 x 10^{-1}	0.10	6.11±0.10 x 10^{-1}
		2.80 x 10^{8}	4.65 x 10^{8}	3.15 x 10^{9}	6.02 x 10^{-1}		
		7.36 x 10^{8}	1.01 x 10^{9}	7.56 x 10^{9}	7.29 x 10^{-1}		
	**Lm_5**	7.42 x 10^{8}	1.10 x 10^{9}	7.54 x 10^{9}	6.75 x 10^{-1}	0.32	6.92±0.32 x 10^{-1}
		7.34 x 10^{8}	1.09 x 10^{9}	7.50 x 10^{9}	6.73 x 10^{-1}		
		1.56 x 10^{8}	1.12 x 10^{9}	7.40 x 10^{8}	1.39 x 10^{-1}		
	**Lm_6**	1.59 x 10^{8}	1.15 x 10^{9}	7.42 x 10^{8}	1.38 x 10^{-1}	0.01	1.38±0.01 x 10^{-1}
		1.59 x 10^{8}	1.16 x 10^{9}	7.46 x 10^{8}	1.37 x 10^{-1}		
**FOS**		1.13 x 10^{9}	3.46 x 10^{9}	2.12 x 10^{9}	3.27 x 10^{-1}		
	**Lm_1**	1.20 x 10^{9}	3.49 x 10^{9}	2.15 x 10^{9}	3.44 x 10^{-1}	0.09	3.36±0.09 x 10^{-1}
		1.15 x 10^{9}	3.40 x 10^{9}	2.17 x 10^{9}	3.38 x 10^{-1}		
		9.28 x 10^{8}	2.62 x 10^{9}	4.40 x 10^{8}	3.54 x 10^{-1}		
	**Lm_2**	9.25 x 10^{8}	2.62 x 10^{9}	4.42 x 10^{8}	3.53 x 10^{-1}	0.05	3.51±0.05 x 10^{-1}
		9.25 x 10^{8}	2.65 x 10^{9}	4.45 x 10^{8}	3.45 x 10^{-1}		
		1.35 x 10^{9}	4.57 x 10^{9}	1.32 x 10^{9}	2.95 x 10^{-1}		
	**Lm_3**	1.40 x 10^{9}	4.59 x 10^{9}	1.38 x 10^{9}	3.05 x 10^{-1}	0.07	3.03±0.07 x 10^{-1}
		1.40 x 10^{9}	4.55 x 10^{9}	1.38 x 10^{9}	3.08 x 10^{-1}		
		1.03 x 10^{9}	4.69 x 10^{9}	3.20 x 10^{9}	2.20 x 10^{-1}		
	**Lm_4**	1.09 x 10^{9}	4.60 x 10^{9}	3.15 x 10^{9}	2.37 x 10^{-1}	0.10	2.31±0.10 x 10^{-1}
		1.09 x 10^{9}	4.62 x 10^{9}	3.15 x 10^{9}	2.36 x 10^{-1}		
		1.07 x 10^{9}	2.18 x 10^{9}	7.56 x 10^{9}	4.91 x 10^{-1}		
	**Lm_5**	1.09 x 10^{9}	2.20 x 10^{9}	7.54 x 10^{9}	4.95 x 10^{-1}	0.02	4.92±0.02 x 10^{-1}
		1.09 x 10^{9}	2.22 x 10^{9}	7.50 x 10^{9}	4.91 x 10^{-1}		
		8.55 x 10^{8}	3.13 x 10^{8}	7.40 x 10^{8}	2.70 x 10^{1}		
	**Lm_6**	8.52 x 10^{8}	3.19 x 10^{8}	7.42 x 10^{8}	2.67 x 10^{1}	0.02	2.68±0.02 x 10^{1}
		8.52 x 10^{8}	3.20 x 10^{8}	7.46 x 10^{8}	2.66 x 10^{1}		
**DA**		7.84 x 10^{8}	2.24 x 10^{9}	2.12 x 10^{9}	3.50 x 10^{-1}		
	**Lm_1**	7.80 x 10^{8}	2.26 x 10^{9}	2.15 x 10^{9}	3.45 x 10^{-1}	0.05	3.43±0.05 x 10^{-1}
		7.80 x 10^{8}	2.29 x 10^{9}	2.17 x 10^{9}	3.40 x 10^{-1}		
		8.72 x 10^{8}	1.67 x 10^{9}	4.40 x 10^{8}	5.22 x 10^{-1}		
	**Lm_2**	8.75 x 10^{8}	1.69 x 10^{9}	4.42 x 10^{8}	5.18 x 10^{-1}	0.03	5.19±0.03 x 10^{-1}
		8.79 x 10^{8}	1.70 x 10^{9}	4.45 x 10^{8}	5.17 x 10^{-1}		
		7.20 x 10^{8}	1.78 x 10^{9}	1.32 x 10^{9}	4.04 x 10^{-1}		
	**Lm_3**	7.20 x 10^{8}	1.75 x 10^{9}	1.38 x 10^{9}	4.11 x 10^{-1}	0.05	4.10±0.05 x 10^{-1}
		7.25 x 10^{8}	1.75 x 10^{9}	1.38 x 10^{9}	4.14 x 10^{-1}		
		9.28 x 10^{8}	2.95 x 10^{9}	3.20 x 10^{9}	3.15 x 10^{-1}		
	**Lm_4**	9.30 x 10^{8}	2.90 x 10^{9}	3.15 x 10^{9}	3.21 x 10^{-1}	0.04	3.17±0.04 x 10^{-1}
		9.30 x 10^{8}	2.96 x 10^{9}	3.15 x 10^{9}	3.14 x 10^{-1}		
		5.64 x 10^{8}	2.98 x 10^{8}	7.56 x 10^{9}	1.90 x 10^{1}		
	**Lm_5**	5.69 x 10^{8}	2.95 x 10^{8}	7.54 x 10^{9}	1.93 x 10^{1}	0.02	1.92±0.02 x 10^{-1}
		5.69 x 10^{8}	2.95 x 10^{8}	7.50 x 10^{9}	1.93 x 10^{1}		
		6.00 x 10^{8}	1.60 x 10^{8}	7.40 x 10^{8}	0.38 x 10^{1}		
	**Lm_6**	6.02 x 10^{8}	1.62 x 10^{8}	7.42 x 10^{8}	0.37 x 10^{1}	0.01	0.38±0.01 x 10^{1}
		6.03 x 10^{8}	1.60 x 10^{8}	7.46 x 10^{8}	0.38 x 10^{1}		

TET – tetracycline, LIN – lincomycin, CIP – ciprofloxacin, FOS – fosfomycin, DA – clindamycin.

Table S5. Antibiotic resistance gene transfer in *Listeria monocytogenes* strains after exposure to HPP **(recovery after 400 MPa)** in microbial culture medium (*in vitro*).

		**T-BHI**	**TD-BHI**	**B-BHI**	**TC/R**	**SD – TC/R**	**Wyniki**
		TC – transconjugants	R – recipient	D - donor	Transfer Rate	±	TC/R±SD
**TET**	**Lm_1**	1.98 x 10^8	1.76 x 10^8	1.23 x 10^9	0.11 x 10^1	0.01	0.11±0.01 x 10^1
		1.89 x 10^8	1.70 x 10^8	1.29 x 10^9	0.11 x 10^1		
		1.95 x 10^8	1.72 x 10^8	1.29 x 10^9	0.13 x 10^1		
	**Lm_2**	5.26 x 10^8	1.71 x 10^8	2.73 x 10^9	0.19 x 10^1	0.07	0.27±0.07 x 10^1
		5.30 x 10^8	1.79 x 10^8	2.73 x 10^9	0.30 x 10^1		
		5.34 x 10^8	1.70 x 10^8	2.76 x 10^9	0.31 x 10^1		
	**Lm_3**	4.66 x 10^8	2.95 x 10^8	1.22 x 10^9	0.16 x 10^1	0.00	0.16±0.00 x 10^1
		4.70 x 10^8	2.90 x 10^8	1.22 x 10^9	0.16 x 10^1		
		4.69 x 10^8	2.96 x 10^8	1.29 x 10^9	0.16 x 10^1		
	**Lm_4**	1.14 x 10^8	3.46 x 10^8	8.32 x 10^8	3.29 x 10^{-1}	0.11	3.40±0.11 x 10^{-1}
		1.16 x 10^8	3.40 x 10^8	8.33 x 10^8	3.41 x 10^{-1}		
		1.20 x 10^8	3.42 x 10^8	8.39 x 10^8	3.51 x 10^{-1}		
	**Lm_5**	3.62 x 10^8	1.58 x 10^8	1.02 x 10^9	0.23 x 10^1	0.01	0.24±0.01 x 10^{-1}
		3.65 x 10^8	1.50 x 10^8	1.09 x 10^9	0.24 x 10^1		
		3.69 x 10^8	1.51 x 10^8	1.02 x 10^9	0.24 x 10^1		
	**Lm_6**	2.21 x 10^8	2.11 x 10^8	2.24 x 10^8	0.11 x 10^1	0.01	0.11±0.01 x 10^{-1}
		2.27 x 10^8	2.10 x 10^8	2.25 x 10^8	0.11 x 10^1		
		2.29 x 10^8	2.19 x 10^8	2.29 x 10^8	0.10 x 10^1		
**LIN**	**Lm_1**	1.98 x 10^8	2.38 x 10^8	1.23 x 10^9	8.32 x 10^{-1}	0.08	8.25±0.08 x 10^{-1}
		1.90 x 10^8	2.30 x 10^8	1.29 x 10^9	8.26 x 10^{-1}		
		1.95 x 10^8	2.39 x 10^8	1.29 x 10^9	8.16 x 10^{-1}		
	**Lm_2**	2.06 x 10^8	5.94 x 10^8	2.73 x 10^9	3.47 x 10^{-1}	0.07	3.51±0.07 x 10^{-1}
		2.12 x 10^8	5.90 x 10^8	2.73 x 10^9	3.59 x 10^{-1}		
		2.08 x 10^8	5.99 x 10^8	2.76 x 10^9	3.47 x 10^{-1}		
	**Lm_3**	2.55 x 10^8	2.51 x 10^8	1.22 x 10^9	0.10 x 10^1	0.00	0.10±0.00 x 10^1
		2.50 x 10^8	2.50 x 10^8	1.22 x 10^9	0.10 x 10^1		
		2.53 x 10^8	2.52 x 10^8	1.29 x 10^9	0.10 x 10^1		
	**Lm_4**	1.99 x 10^8	1.92 x 10^8	8.32 x 10^8	0.10 x 10^1	0.00	0.10±0.00 x 10^1
		1.90 x 10^8	1.90 x 10^8	8.33 x 10^8	0.10 x 10^1		
		1.95 x 10^8	1.94 x 10^8	8.39 x 10^8	0.10 x 10^1		
	**Lm_5**	1.69 x 10^8	2.18 x 10^8	1.02 x 10^9	7.75 x 10^{-1}	0.17	7.71±0.17 x 10^{-1}
		1.65 x 10^8	2.10 x 10^8	1.09 x 10^9	7.86 x 10^{-1}		
		1.62 x 10^8	2.15 x 10^8	1.02 x 10^9	7.53 x 10^{-1}		
	**Lm_6**	2.18 x 10^8	2.08 x 10^8	2.24 x 10^8	0.11 x 10^1	0.01	0.11±0.01 x 10^1
		2.15 x 10^8	2.10 x 10^8	2.25 x 10^8	0.10 x 10^1		
		2.10 x 10^8	2.12 x 10^8	2.29 x 10^8	9.90 x 10^{-1}		
**CIP**	**Lm_1**	1.78 x 10^8	1.27 x 10^8	1.23 x 10^9	0.14 x 10^1	0.00	0.14±0.00 x 10^1
		1.75 x 10^8	1.29 x 10^8	1.29 x 10^9	0.14 x 10^1		
		1.70 x 10^8	1.23 x 10^8	1.29 x 10^9	0.14 x 10^1		
	**Lm_2**	1.20 x 10^8	1.87 x 10^8	2.73 x 10^9	6.42 x 10^{-1}	0.30	6.76±0.30 x 10^{-1}
		1.25 x 10^8	1.80 x 10^8	2.73 x 10^9	6.94 x 10^{-1}		
		1.29 x 10^8	1.86 x 10^8	2.76 x 10^9	6.93 x 10^{-1}		
	**Lm_3**	1.06 x 10^8	1.26 x 10^8	1.22 x 10^9	8.41 x 10^{-1}	0.39	8.73±0.39 x 10^{-1}
		1.10 x 10^8	1.20 x 10^8	1.22 x 10^9	9.17 x 10^{-1}		
		1.05 x 10^8	1.22 x 10^8	1.29 x 10^9	8.61 x 10^{-1}		

		1.38×10^{8}	9.36×10^{7}	8.32×10^{8}	1.47×10^{-1}		
	**Lm_4**	1.42×10^{8}	9.33×10^{7}	8.33×10^{8}	1.52×10^{-1}	0.04	$1.51 \pm 0.04 \times 10^{-1}$
		1.45×10^{8}	9.39×10^{7}	8.39×10^{8}	1.54×10^{-1}		
		1.54×10^{8}	1.14×10^{8}	1.02×10^{9}	0.14×10^{1}		
	**Lm_5**	1.50×10^{8}	1.10×10^{8}	1.09×10^{9}	0.14×10^{1}	0.01	$0.14 \pm 0.01 \times 10^{1}$
		1.52×10^{8}	1.15×10^{8}	1.02×10^{9}	0.13×10^{1}		
		3.04×10^{8}	6.72×10^{7}	2.24×10^{8}	4.52×10^{-1}		
	**Lm_6**	3.10×10^{8}	6.70×10^{7}	2.25×10^{8}	4.62×10^{-1}	0.06	$4.59 \pm 0.06 \times 10^{-1}$
		3.12×10^{8}	6.75×10^{7}	2.29×10^{8}	4.62×10^{-1}		
**FOS**		2.50×10^{8}	2.24×10^{8}	1.23×10^{9}	0.11×10^{1}		
	**Lm_1**	2.55×10^{8}	2.25×10^{8}	1.29×10^{9}	0.11×10^{1}	0.00	$0.11 \pm 0.00 \times 10^{1}$
		2.54×10^{8}	2.29×10^{8}	1.29×10^{9}	0.11×10^{1}		
		5.11×10^{8}	4.30×10^{8}	2.73×10^{9}	0.12×10^{1}		
	**Lm_2**	5.16×10^{8}	4.35×10^{8}	2.73×10^{9}	0.12×10^{1}	0.00	$0.12 \pm 0.00 \times 10^{1}$
		5.18×10^{8}	4.39×10^{8}	2.76×10^{9}	0.12×10^{1}		
		3.30×10^{8}	2.26×10^{8}	1.22×10^{9}	0.15×10^{1}		
	**Lm_3**	3.34×10^{8}	2.27×10^{8}	1.22×10^{9}	0.15×10^{1}	0.00	$0.15 \pm 0.00 \times 10^{1}$
		3.39×10^{8}	2.29×10^{8}	1.29×10^{9}	0.15×10^{1}		
		1.84×10^{8}	1.62×10^{8}	8.32×10^{8}	0.11×10^{1}		
	**Lm_4**	1.89×10^{8}	1.57×10^{8}	8.33×10^{8}	0.12×10^{1}	0.01	$0.12 \pm 0.01 \times 10^{1}$
		1.84×10^{8}	1.60×10^{8}	8.39×10^{8}	0.12×10^{1}		
		1.88×10^{8}	1.94×10^{8}	1.02×10^{9}	9.69×10^{-1}		
	**Lm_5**	1.80×10^{8}	1.90×10^{8}	1.09×10^{9}	9.47×10^{-1}	0.20	$9.49 \pm 0.20 \times 10^{-1}$
		1.85×10^{8}	1.99×10^{8}	1.02×10^{9}	9.30×10^{-1}		
		3.05×10^{8}	1.78×10^{8}	2.24×10^{8}	0.17×10^{1}		
	**Lm_6**	3.09×10^{8}	1.70×10^{8}	2.25×10^{8}	0.18×10^{1}	0.01	$0.17 \pm 0.01 \times 10^{1}$
		3.00×10^{8}	1.75×10^{8}	2.29×10^{8}	0.17×10^{1}		
**DA**		1.73×10^{8}	1.87×10^{8}	1.23×10^{9}	9.25×10^{-1}		
	**Lm_1**	1.79×10^{8}	1.80×10^{8}	1.29×10^{9}	9.94×10^{-1}	0.36	$9.54 \pm 0.36 \times 10^{-1}$
		1.78×10^{8}	1.89×10^{8}	1.29×10^{9}	9.42×10^{-1}		
		1.54×10^{8}	4.88×10^{8}	2.73×10^{9}	3.16×10^{-1}		
	**Lm_2**	1.59×10^{8}	4.90×10^{8}	2.73×10^{9}	3.24×10^{-1}	0.10	$3.15 \pm 0.10 \times 10^{-1}$
		1.50×10^{8}	4.92×10^{8}	2.76×10^{9}	3.05×10^{-1}		
		1.75×10^{8}	1.73×10^{8}	1.22×10^{9}	0.10×10^{1}		
	**Lm_3**	1.79×10^{8}	1.70×10^{8}	1.22×10^{9}	0.11×10^{1}	0.51	$0.40 \pm 0.51 \times 10^{1}$
		1.78×10^{8}	1.79×10^{8}	1.29×10^{9}	9.94×10^{-1}		
		2.26×10^{8}	2.51×10^{8}	8.32×10^{8}	9.00×10^{-1}		
	**Lm_4**	2.29×10^{8}	2.59×10^{8}	8.33×10^{8}	8.84×10^{-1}	0.09	$8.94 \pm 0.09 \times 10^{-1}$
		2.29×10^{8}	2.55×10^{8}	8.39×10^{8}	8.98×10^{-1}		
		1.27×10^{8}	1.46×10^{8}	1.02×10^{9}	8.70×10^{-1}		
	**Lm_5**	1.20×10^{8}	1.40×10^{8}	1.09×10^{9}	8.57×10^{-1}	0.19	$8.53 \pm 0.19 \times 10^{-1}$
		1.24×10^{8}	1.49×10^{8}	1.02×10^{9}	8.32×10^{-1}		
		1.87×10^{8}	2.10×10^{8}	2.24×10^{8}	8.90×10^{-1}		
	**Lm_6**	1.80×10^{8}	2.15×10^{8}	2.25×10^{8}	8.37×10^{-1}	0.30	$8.56 \pm 0.30 \times 10^{-1}$
		1.84×10^{8}	2.19×10^{8}	2.29×10^{8}	8.40×10^{-1}		

TET – tetracycline, LIN – lincomycin, CIP – ciprofloxacin, FOS – fosfomycin, DA – clindamycin.

Table S6. Antibiotic resistance gene transfer in *Listeria monocytogenes* strains after exposure to HPP (**200 MPa)** in food matrix (*in situ*).

		**T-BHI**	**TD-BHI**	**B-BHI**	**TC/R**	**SD – TC/R**	**Wyniki**
		TC – transconjugants	R – recipient	D - donor	Transfer Rate	±	TC/R±SD
**TET**	**Lm_1**	9.78×10^{8}	2.00×10^{9}	2.12×10^{9}	4.89×10^{-1}	0.14	$4.74 \pm 0.14 \times 10^{-1}$
		9.70×10^{8}	2.10×10^{9}	2.19×10^{9}	4.62×10^{-1}		
		9.75×10^{8}	2.07×10^{9}	2.12×10^{9}	4.71×10^{-1}		
	**Lm_2**	5.52×10^{8}	2.20×10^{8}	4.40×10^{8}	0.25×10^{1}	0.01	$0.25 \pm 0.01 \times 10^{1}$
		5.49×10^{8}	2.22×10^{8}	4.41×10^{8}	0.25×10^{1}		
		5.55×10^{8}	2.29×10^{8}	4.49×10^{8}	0.24×10^{1}		
	**Lm_3**	3.80×10^{8}	1.07×10^{9}	1.32×10^{9}	3.55×10^{-1}	0.16	$3.55 \pm 0.16 \times 10^{-1}$
		3.80×10^{8}	1.12×10^{9}	1.39×10^{9}	3.39×10^{-1}		
		3.82×10^{8}	1.03×10^{9}	1.32×10^{9}	3.71×10^{-1}		
	**Lm_4**	3.36×10^{8}	2.60×10^{9}	3.20×10^{9}	1.29×10^{-1}	0.02	$1.31 \pm 0.02 \times 10^{-1}$
		3.40×10^{8}	2.55×10^{9}	3.21×10^{9}	1.33×10^{-1}		
		3.42×10^{8}	2.62×10^{9}	3.29×10^{9}	1.31×10^{-1}		
	**Lm_5**	6.56×10^{8}	1.26×10^{10}	7.56×10^{9}	5.21×10^{-2}	0.16	$5.39 \pm 0.16 \times 10^{-2}$
		6.60×10^{8}	1.20×10^{10}	7.59×10^{9}	5.50×10^{-2}		
		6.60×10^{8}	1.21×10^{10}	7.50×10^{9}	5.45×10^{-2}		
	**Lm_6**	4.80×10^{8}	6.00×10^{8}	7.40×10^{8}	8.00×10^{-1}	0.05	$8.03 \pm 0.05 \times 10^{-1}$
		4.85×10^{8}	6.00×10^{8}	7.41×10^{8}	8.08×10^{-1}		
		4.88×10^{8}	6.10×10^{8}	7.49×10^{8}	8.00×10^{-1}		
**LIN**	**Lm_1**	5.52×10^{8}	1.83×10^{9}	2.12×10^{9}	3.01×10^{-1}	0.06	$2.94 \pm 0.06 \times 10^{-1}$
		5.50×10^{8}	1.89×10^{9}	2.19×10^{9}	2.91×10^{-1}		
		5.50×10^{8}	1.89×10^{9}	2.12×10^{9}	2.91×10^{-1}		
	**Lm_2**	1.48×10^{8}	3.70×10^{8}	4.40×10^{8}	4.00×10^{-1}	0.05	$3.95 \pm 0.05 \times 10^{-1}$
		1.45×10^{8}	3.72×10^{8}	4.41×10^{8}	3.90×10^{-1}		
		1.50×10^{8}	3.79×10^{8}	4.49×10^{8}	3.96×10^{-1}		
	**Lm_3**	3.88×10^{8}	1.95×10^{9}	1.32×10^{9}	1.99×10^{-1}	0.03	$2.02 \pm 0.03 \times 10^{-1}$
		3.88×10^{8}	1.92×10^{9}	1.39×10^{9}	2.02×10^{-1}		
		3.90×10^{8}	1.90×10^{9}	1.32×10^{9}	2.05×10^{-1}		
	**Lm_4**	6.68×10^{8}	2.08×10^{9}	3.20×10^{9}	3.21×10^{-1}	0.04	$3.18 \pm 0.04 \times 10^{-1}$
		6.65×10^{8}	2.08×10^{9}	3.21×10^{9}	3.20×10^{-1}		
		6.65×10^{8}	2.12×10^{9}	3.29×10^{9}	3.14×10^{-1}		
	**Lm_5**	1.24×10^{9}	5.93×10^{9}	7.56×10^{9}	2.10×10^{-1}	0.04	$2.05 \pm 0.04 \times 10^{-1}$
		1.20×10^{9}	5.90×10^{9}	7.59×10^{9}	2.03×10^{-1}		
		1.20×10^{9}	5.95×10^{9}	7.50×10^{9}	2.02×10^{-1}		
	**Lm_6**	2.52×10^{8}	3.60×10^{8}	7.40×10^{8}	7.00×10^{-1}	0.07	$6.98 \pm 0.07 \times 10^{-1}$
		2.55×10^{8}	3.62×10^{8}	7.41×10^{8}	7.04×10^{-1}		
		2.55×10^{8}	3.69×10^{8}	7.49×10^{8}	6.91×10^{-1}		
**CIP**	**Lm_1**	5.46×10^{8}	1.60×10^{8}	2.12×10^{9}	0.34×10^{1}	0.01	$0.34 \pm 0.01 \times 10^{1}$
		5.50×10^{8}	1.62×10^{8}	2.19×10^{9}	0.34×10^{1}		
		5.50×10^{8}	1.69×10^{8}	2.12×10^{9}	0.33×10^{1}		
	**Lm_2**	2.60×10^{8}	9.92×10^{7}	4.40×10^{8}	0.26×10^{1}	0.01	$0.27 \pm 0.01 \times 10^{1}$
		2.65×10^{8}	9.89×10^{7}	4.41×10^{8}	0.27×10^{1}		
		2.65×10^{8}	9.80×10^{7}	4.49×10^{8}	0.27×10^{1}		
	**Lm_3**	2.08×10^{8}	7.80×10^{8}	1.32×10^{9}	2.70×10^{-1}	0.08	$2.65 \pm 0.08 \times 10^{-1}$
		2.10×10^{8}	7.85×10^{8}	1.39×10^{9}	2.55×10^{-1}		
		2.10×10^{8}	7.80×10^{8}	1.32×10^{9}	2.69×10^{-1}		

	**Lm_4**	2.80×10^{8}	4.60×10^{8}	3.20×10^{9}	6.10×10^{-1}	0.06	$6.16 \pm 0.06 \times 10^{-1}$
		2.85×10^{8}	4.62×10^{8}	3.21×10^{9}	6.17×10^{-1}		
		2.85×10^{8}	4.59×10^{8}	3.29×10^{9}	6.21×10^{-1}		
	**Lm_5**	7.36×10^{8}	1.01×10^{9}	7.56×10^{9}	7.29×10^{-1}	0.19	$7.08 \pm 0.19 \times 10^{-1}$
		7.40×10^{8}	1.06×10^{9}	7.59×10^{9}	6.98×10^{-1}		
		7.38×10^{8}	1.06×10^{9}	7.50×10^{9}	6.96×10^{-1}		
	**Lm_6**	1.56×10^{8}	1.12×10^{9}	7.40×10^{8}	1.39×10^{-1}	0.03	$1.37 \pm 0.03 \times 10^{-1}$
		1.59×10^{8}	1.19×10^{9}	7.41×10^{8}	1.34×10^{-1}		
		1.56×10^{8}	1.12×10^{9}	7.49×10^{8}	1.39×10^{-1}		
**FOS**	**Lm_1**	1.13×10^{9}	3.46×10^{9}	2.72×10^{9}	3.27×10^{-1}	0.09	$3.34 \pm 0.09 \times 10^{-1}$
		1.15×10^{9}	3.47×10^{9}	2.79×10^{9}	3.31×10^{-1}		
		1.20×10^{9}	3.49×10^{9}	2.70×10^{9}	3.44×10^{-1}		
	**Lm_2**	9.28×10^{8}	2.62×10^{9}	2.39×10^{9}	3.54×10^{-1}	0.05	$3.51 \pm 0.05 \times 10^{-1}$
		9.28×10^{8}	2.69×10^{9}	2.39×10^{9}	3.45×10^{-1}		
		9.25×10^{8}	2.62×10^{9}	2.30×10^{9}	3.53×10^{-1}		
	**Lm_3**	1.35×10^{9}	4.57×10^{9}	2.34×10^{9}	2.95×10^{-1}	0.28	$2.83 \pm 0.28 \times 10^{-1}$
		1.38×10^{9}	4.57×10^{9}	2.35×10^{9}	3.02×10^{-1}		
		1.38×10^{9}	5.50×10^{9}	2.35×10^{9}	2.51×10^{-1}		
	**Lm_4**	1.03×10^{9}	4.69×10^{9}	3.38×10^{9}	2.20×10^{-1}	0.08	$2.29 \pm 0.08 \times 10^{-1}$
		1.09×10^{9}	4.70×10^{9}	3.39×10^{9}	2.32×10^{-1}		
		1.09×10^{9}	4.65×10^{9}	3.40×10^{9}	2.34×10^{-1}		
	**Lm_5**	1.07×10^{9}	2.18×10^{9}	2.10×10^{9}	4.91×10^{-1}	0.19	$5.09 \pm 0.19 \times 10^{-1}$
		1.11×10^{9}	2.19×10^{9}	2.14×10^{9}	5.07×10^{-1}		
		1.11×10^{9}	2.10×10^{9}	2.14×10^{9}	5.29×10^{-1}		
	**Lm_6**	8.55×10^{8}	3.13×10^{8}	1.83×10^{9}	0.27×10^{1}	0.00	$0.27 \pm 0.00 \times 10^{-1}$
		8.55×10^{8}	3.12×10^{8}	1.89×10^{9}	0.27×10^{1}		
		8.50×10^{8}	3.19×10^{8}	1.83×10^{9}	0.27×10^{1}		
**DA**	**Lm_1**	7.84×10^{8}	2.24×10^{9}	2.12×10^{9}	3.50×10^{-1}	0.03	$3.49 \pm 0.03 \times 10^{-1}$
		7.90×10^{8}	2.25×10^{9}	2.19×10^{9}	3.51×10^{-1}		
		7.90×10^{8}	2.29×10^{9}	2.12×10^{9}	3.45×10^{-1}		
	**Lm_2**	8.72×10^{8}	1.67×10^{9}	4.40×10^{8}	5.22×10^{-1}	0.05	$5.23 \pm 0.05 \times 10^{-1}$
		8.72×10^{8}	1.65×10^{9}	4.41×10^{8}	5.28×10^{-1}		
		8.80×10^{8}	1.70×10^{9}	4.49×10^{8}	5.18×10^{-1}		
	**Lm_3**	7.20×10^{8}	1.78×10^{9}	1.32×10^{9}	4.04×10^{-1}	0.02	$4.05 \pm 0.02 \times 10^{-1}$
		7.25×10^{8}	1.78×10^{9}	1.39×10^{9}	4.07×10^{-1}		
		7.25×10^{8}	1.79×10^{9}	1.32×10^{9}	4.05×10^{-1}		
	**Lm_4**	9.28×10^{8}	2.95×10^{9}	3.20×10^{9}	3.15×10^{-1}	0.03	$3.17 \pm 0.03 \times 10^{-1}$
		9.30×10^{8}	2.90×10^{9}	3.21×10^{9}	3.21×10^{-1}		
		9.30×10^{8}	2.95×10^{9}	3.29×10^{9}	3.15×10^{-1}		
	**Lm_5**	5.64×10^{8}	2.98×10^{8}	7.56×10^{9}	0.19×10^{1}	0.00	$0.19 \pm 0.00 \times 10^{-1}$
		5.60×10^{8}	2.90×10^{8}	7.59×10^{9}	0.19×10^{1}		
		5.61×10^{8}	2.92×10^{8}	7.50×10^{9}	0.19×10^{1}		
	**Lm_6**	6.00×10^{8}	1.60×10^{8}	7.40×10^{8}	0.38×10^{1}	0.01	$0.37 \pm 0.01 \times 10^{1}$
		6.09×10^{8}	1.63×10^{8}	7.41×10^{8}	0.37×10^{1}		
		6.05×10^{8}	1.69×10^{8}	7.49×10^{8}	0.36×10^{1}		

TET – tetracycline, LIN – lincomycin, CIP – ciprofloxacin, FOS – fosfomycin, DA – clindamycin.

Table S7. Antibiotic resistance gene transfer in *Listeria monocytogenes* strains after exposure to HPP (**recovery after 400 MPa)** in food matrix (*in situ*).

		**T-BHI**	**TD-BHI**	**B-BHI**	**TC/R**	**SD – TC/R**	**Wyniki**
		**TC – transconjugants**	**R – recipient**	**D - donor**	**Transfer Rate**	±	TC/R±SD
		1.15 x 10^{8}	7.70 x 10^{7}	3.20 x 10^{8}	1.49 x 10^{2}		
	**Lm_1**	1.19 x 10^{8}	7.75 x 10^{7}	3.20 x 10^{8}	1.54 x 10^{2}	0.03	1.51±0.03 x 10^{2}
		1.17 x 10^{8}	7.79 x 10^{7}	3.28 x 10^{8}	1.50 x 10^{2}		
		1.35 x 10^{8}	1.16 x 10^{8}	1.50 x 10^{8}	0.11 x 10^{1}		
	**Lm_2**	1.39 x 10^{8}	1.19 x 10^{8}	1.51 x 10^{8}	0.12 x 10^{1}	0.01	0.12±0.01 x 10^{1}
		1.35 x 10^{8}	1.10 x 10^{8}	1.50 x 10^{8}	0.12 x 10^{1}		
		9.60 x 10^{7}	1.32 x 10^{8}	1.70 x 10^{8}	0.72 x 10^{-1}		
	**Lm_3**	9.66 x 10^{7}	1.39 x 10^{8}	1.76 x 10^{8}	0.69 x 10^{-1}	0.02	0.70±0.02 x 10^{-1}
		9.69 x 10^{7}	1.39 x 10^{8}	1.76 x 10^{8}	0.70 x 10^{-1}		
**TET**		1.15 x 10^{8}	2.80 x 10^{8}	1.50 x 10^{8}	0.41 x 10^{1}		
	**Lm_4**	1.19 x 10^{8}	2.86 x 10^{8}	1.53 x 10^{8}	0.42 x 10^{1}	0.02	0.41±0.02 x 10^{1}
		1.10 x 10^{8}	2.80 x 10^{8}	1.54 x 10^{8}	0.39 x 10^{1}		
		1.08 x 10^{8}	8.60 x 10^{7}	1.10 x 10^{8}	0.13 x 10^{1}		
	**Lm_5**	1.09 x 10^{8}	8.65 x 10^{7}	1.16 x 10^{8}	0.13 x 10^{1}	0.01	0.13±0.01 x 10^{1}
		1.01 x 10^{8}	8.65 x 10^{7}	1.15 x 10^{8}	0.12 x 10^{1}		
		9.60 x 10^{8}	1.80 x 10^{8}	2.70 x 10^{8}	0.53 x 10^{1}		
	**Lm_6**	9.65 x 10^{8}	1.89 x 10^{8}	2.75 x 10^{8}	0.51 x 10^{1}	0.01	0.52±0.01 x 10^{1}
		9.66 x 10^{8}	1.89 x 10^{8}	2.78 x 10^{8}	0.51 x 10^{1}		
		1.50 x 10^{8}	9.10 x 10^{7}	3.20 x 10^{8}	0.19 x 10^{1}		
	**Lm_1**	1.59 x 10^{8}	9.10 x 10^{7}	3.20 x 10^{8}	0.17 x 10^{1}	0.01	0.18±0.01 x 10^{1}
		1.56 x 10^{8}	9.04 x 10^{7}	3.28 x 10^{8}	0.17 x 10^{1}		
		6.70 x 10^{7}	9.80 x 10^{7}	1.50 x 10^{8}	6.83 x 10^{-1}		
	**Lm_2**	6.75 x 10^{7}	9.80 x 10^{7}	1.51 x 10^{8}	6.89 x 10^{-1}	0.10	6.81±0.10 x 10^{-1}
		6.70 x 10^{7}	9.99 x 10^{7}	1.50 x 10^{8}	6.70 x 10^{-1}		
		6.90 x 10^{7}	2.03 x 10^{8}	1.70 x 10^{8}	3.40 x 10^{-1}		
	**Lm_3**	6.99 x 10^{7}	2.03 x 10^{8}	1.76 x 10^{8}	3.44 x 10^{-1}	0.07	3.38±0.07 x 10^{-1}
		6.90 x 10^{7}	2.09 x 10^{8}	1.76 x 10^{8}	3.30 x 10^{-1}		
**LIN**		2.17 x 10^{8}	1.08 x 10^{8}	1.50 x 10^{8}	0.20 x 10^{1}		
	**Lm_4**	2.19 x 10^{8}	1.14 x 10^{8}	1.53 x 10^{8}	0.19 x 10^{1}	0.01	0.19±0.01 x 10^{1}
		2.20 x 10^{8}	1.14 x 10^{8}	1.54 x 10^{8}	0.19 x 10^{1}		
		1.25 x 10^{8}	1.29 x 10^{8}	1.10 x 10^{8}	9.69 x 10^{-1}		
	**Lm_5**	1.29 x 10^{8}	1.23 x 10^{8}	1.16 x 10^{8}	0.10 x 10^{1}	0.00	0.10±0.00 x 10^{-1}
		1.29 x 10^{8}	1.23 x 10^{8}	1.15 x 10^{8}	0.10 x 10^{1}		
		1.38 x 10^{8}	1.11 x 10^{8}	2.70 x 10^{8}	0.12 x 10^{-1}		
	**Lm_6**	1.39 x 10^{8}	1.18 x 10^{8}	2.75 x 10^{8}	0.12 x 10^{-1}	0.00	0.12±0.00 x 10^{-1}
		1.39 x 10^{8}	1.18 x 10^{8}	2.78 x 10^{8}	0.12 x 10^{-1}		
		5.10 x 10^{7}	6.00 x 10^{5}	3.20 x 10^{8}	8.50 x 10^{1}		
	**Lm_1**	5.19 x 10^{7}	6.05 x 10^{5}	3.20 x 10^{8}	8.58 x 10^{1}	0.06	8.51±0.06 x 10^{1}
		5.15 x 10^{7}	6.09 x 10^{5}	3.28 x 10^{8}	8.46 x 10^{1}		
		5.80 x 10^{7}	9.00 x 10^{5}	1.50 x 10^{8}	6.44 x 10^{1}		
**CIP**	**Lm_2**	5.88 x 10^{7}	9.09 x 10^{5}	1.51 x 10^{8}	6.46 x 10^{1}	0.02	6.46±0.02 x 10^{1}
		5.88 x 10^{7}	9.08 x 10^{5}	1.50 x 10^{8}	6.48 x 10^{1}		
		2.90 x 10^{7}	1.10 x 10^{6}	1.70 x 10^{8}	2.63 x 10^{1}		
	**Lm_3**	2.90 x 10^{7}	1.19 x 10^{6}	1.76 x 10^{8}	2.44 x 10^{1}	0.10	2.55±0.10 x 10^{1}
		2.95 x 10^{7}	1.15 x 10^{6}	1.76 x 10^{8}	2.57 x 10^{1}		

		1.08×10^{8}	7.40×10^{5}	1.50×10^{8}	1.46×10^{2}		
	**Lm_4**	1.11×10^{8}	7.43×10^{5}	1.53×10^{8}	1.49×10^{2}	0.02	$1.48 \pm 0.02 \times 10^{2}$
		1.11×10^{8}	7.48×10^{5}	1.54×10^{8}	1.48×10^{2}		
		4.90×10^{7}	8.20×10^{5}	1.10×10^{8}	5.98×10^{1}		
	**Lm_5**	4.94×10^{7}	8.24×10^{5}	1.16×10^{8}	5.98×10^{1}	0.01	$5.99 \pm 0.01 \times 10^{1}$
		4.99×10^{7}	8.29×10^{5}	1.15×10^{8}	6.00×10^{1}		
		5.90×10^{7}	1.00×10^{5}	2.70×10^{8}	5.90×10^{2}		
	**Lm_6**	5.95×10^{7}	1.08×10^{5}	2.75×10^{8}	5.51×10^{2}	0.21	$5.65 \pm 0.21 \times 10^{2}$
		5.99×10^{7}	1.08×10^{5}	2.78×10^{8}	5.55×10^{2}		
**FOS**		1.51×10^{8}	2.05×10^{8}	3.20×10^{8}	7.37×10^{-1}		
	**Lm_1**	1.59×10^{8}	2.09×10^{8}	3.20×10^{8}	7.61×10^{-1}	0.13	$7.47 \pm 0.13 \times 10^{-1}$
		1.55×10^{8}	2.09×10^{8}	3.28×10^{8}	7.42×10^{-1}		
		1.35×10^{8}	1.50×10^{8}	1.50×10^{8}	9.00×10^{-1}		
	**Lm_2**	1.37×10^{8}	1.54×10^{8}	1.51×10^{8}	8.90×10^{-1}	0.20	$8.84 \pm 0.20 \times 10^{-1}$
		1.37×10^{8}	1.59×10^{8}	1.50×10^{8}	8.62×10^{-1}		
		8.60×10^{7}	1.75×10^{8}	1.70×10^{8}	4.91×10^{-1}		
	**Lm_3**	8.64×10^{7}	1.77×10^{8}	1.76×10^{8}	4.88×10^{-1}	0.04	$4.87 \pm 0.04 \times 10^{-1}$
		8.64×10^{7}	1.79×10^{8}	1.76×10^{8}	4.83×10^{-1}		
		2.28×10^{8}	2.36×10^{8}	1.50×10^{8}	9.66×10^{-1}		
	**Lm_4**	2.32×10^{8}	2.39×10^{8}	1.53×10^{8}	9.71×10^{-1}	0.02	$0.98 \pm 0.02 \times 10^{-1}$
		2.32×10^{8}	2.30×10^{8}	1.54×10^{8}	0.10×10^{1}		
		1.42×10^{8}	2.04×10^{8}	1.10×10^{8}	6.96×10^{-1}		
	**Lm_5**	1.49×10^{8}	2.05×10^{8}	1.16×10^{8}	7.27×10^{-1}	0.18	$7.17 \pm 0.18 \times 10^{-1}$
		1.49×10^{8}	2.05×10^{8}	1.15×10^{8}	7.27×10^{-1}		
		1.36×10^{8}	1.84×10^{8}	2.70×10^{8}	7.39×10^{-1}		
	**Lm_6**	1.39×10^{8}	1.89×10^{8}	2.75×10^{8}	7.35×10^{-1}	0.02	$7.36 \pm 0.02 \times 10^{-1}$
		1.39×10^{8}	1.89×10^{8}	2.78×10^{8}	7.35×10^{-1}		
**DA**		8.10×10^{7}	8.00×10^{7}	3.20×10^{8}	0.10×10^{-1}		
	**Lm_1**	8.19×10^{7}	8.01×10^{7}	3.20×10^{8}	0.10×10^{-1}	0.00	$0.10 \pm 0.00 \times 10^{-1}$
		8.19×10^{7}	8.05×10^{7}	3.28×10^{8}	0.10×10^{-1}		
		5.30×10^{7}	8.30×10^{7}	1.50×10^{8}	6.39×10^{-1}		
	**Lm_2**	5.38×10^{7}	8.30×10^{7}	1.51×10^{8}	6.48×10^{-1}	0.05	$6.43 \pm 0.05 \times 10^{-1}$
		5.39×10^{7}	8.39×10^{7}	1.50×10^{8}	6.42×10^{-1}		
		7.50×10^{7}	1.34×10^{8}	1.70×10^{8}	5.60×10^{-1}		
	**Lm_3**	7.50×10^{7}	1.35×10^{8}	1.76×10^{8}	5.56×10^{-1}	0.07	$5.54 \pm 0.07 \times 10^{-1}$
		7.59×10^{7}	1.39×10^{8}	1.76×10^{8}	5.46×10^{-1}		
		1.26×10^{8}	8.00×10^{7}	1.50×10^{8}	0.16×10^{1}		
	**Lm_4**	1.29×10^{8}	8.07×10^{7}	1.53×10^{8}	0.16×10^{1}	0.00	$0.16 \pm 0.00 \times 10^{1}$
		1.29×10^{8}	8.01×10^{7}	1.54×10^{8}	0.16×10^{1}		
		9.50×10^{7}	7.70×10^{7}	1.10×10^{8}	0.12×10^{-1}		
	**Lm_5**	9.55×10^{7}	7.72×10^{7}	1.16×10^{8}	0.12×10^{-1}	0.00	$0.12 \pm 0.00 \times 10^{-1}$
		9.55×10^{7}	7.74×10^{7}	1.15×10^{8}	0.12×10^{-1}		
		6.40×10^{7}	7.80×10^{7}	2.70×10^{8}	8.21×10^{-1}		
	**Lm_6**	6.45×10^{7}	7.89×10^{7}	2.75×10^{8}	8.17×10^{-1}	0.05	$8.22 \pm 0.05 \times 10^{-1}$
		6.45×10^{7}	7.80×10^{7}	2.78×10^{8}	8.27×10^{-1}		

TET – tetracycline, LIN – lincomycin, CIP – ciprofloxacin, FOS – fosfomycin, DA – clindamycin.

Statistical tests used in the research.

Table S8. Analyze differences in gene transfer frequencies between different pressure conditions – *p* value < 0.05 (Friedman's ANOVA test).

**Antibiotic**	**Conditions**	
	in vitro	***in situ***
**TET**	0.311403	0.135335
**LIN**	0.069483	0.606531
**CIP**	0.833753	0.002479
**FOS**	0.114559	0.311403
**DA**	0.606531	0.846482

TET – tetracycline, LIN – lincomycin, CIP – ciprofloxacin, FOS – fosfomycin, DA – clindamycin.

Table S9. Analyze differences in the effect of pressure on resistance gene transfer – *p* value < 0.05 (Friedman's ANOVA test).

**Conditions**	**Antibiotic**				
	**TET**	**LIN**	**CIP**	**FOS**	**DA**
**Control (*in vitro*)**	0.401678	0.036032	1.000000	0.401678	1.000000
**200 MPa (*in vitro*)**	0.401678	0.036032	1.000000	0.401678	0.401678
**Recovery after 400 MPa (*in vitro*)**	0.833935	0.201243	0.528233	0.058475	0.401678
**Control (*in situ*)**	0.035522	0.036032	0.036032	0.401678	0.059058
**200 MPa (*in situ*)**	0.401678	0.036032	1.000000	0.036032	0.401678
**Recovery after 400 MPa (*in situ*)**	0.247561	0.529368	0.036032	0.036032	0.208413

TET – tetracycline, LIN – lincomycin, CIP – ciprofloxacin, FOS – fosfomycin, DA – clindamycin. Control – control sample (transconjugant obtained from the strain before HPP treatment); 200 MPa – transconjugant obtained from the strain after exposure to 200 MPa pressure; 400 MPa – transconjugant obtained from the recovered strain after exposure to 400 MPa pressure.

Table S10. The frequency of resistance gene transfer comparisons between different antibiotics under the same conditions – p value < 0.05 (Kruskal-Wallis test).

**Conditions**	**p value**
**Control (*in vitro*)**	0.509103
**200 MPa (*in vitro*)**	0.799615
**Recovery after 400 MPa (*in vitro*)**	0.330772
**Control (*in situ*)**	0.012698
**200 MPa (*in situ*)**	0.553158
**Recovery after 400 MPa (*in situ*)**	0.003999

Control – control sample (transconjugant obtained from the strain before HPP treatment); 200 MPa – transconjugant obtained from the strain after exposure to 200 MPa pressure; 400 MPa – transconjugant obtained from the recovered strain after exposure to 400 MPa pressure.

Table S11. Assessment of the strength and direction of the relationship between pressure conditions and the frequency of gene transfer for each antibiotic – p value < 0.05 (Spearman correlation).

Conditions	Antibiotic				
	**TET**	**LIN**	**CIP**	**FOS**	**DA**
**200 MPa**	NA	NA	NA	0.787172	NA
**Recovery after 400 MPa**	0.328723	1.00000	0.199660	0.826848	0.704000

TET – tetracycline, LIN – lincomycin, CIP – ciprofloxacin, FOS – fosfomycin, DA – clindamycin. 200 MPa – transconjugant obtained from the strain after exposure to 200 MPa pressure; 400 MPa – transconjugant obtained from the recovered strain after exposure to 400 MPa pressure.

Table S12. Assessment of the strength and direction of the relationship between pressure conditions and the frequency of gene transfer – p value < 0.05 (Spearman correlation).

Conditions	p values
**200 MPa**	< 0.000001
**Recovery after 400 MPa**	0.143678
